# Methylome-Wide Association Study in Peripheral White Blood Cells Focusing on Central Obesity and Inflammation

**DOI:** 10.3390/genes10060444

**Published:** 2019-06-11

**Authors:** Ana Arpón, Fermín I. Milagro, Omar Ramos-Lopez, Maria L. Mansego, José-Ignacio Riezu-Boj, J. Alfredo Martínez

**Affiliations:** 1 Department of Nutrition, Food Sciences and Physiology, University of Navarra, Irunlarrea 1, 31008 Pamplona, Spain; aarpon.1@alumni.unav.es (A.A.); fmilagro@unav.es (F.I.M.); os_mar6@hotmail.com (O.R.-L.); jalfmtz@unav.es (J.A.M.); 2 Centre for Nutrition Research, University of Navarra, Irunlarrea 1, 31008 Pamplona, Spain; 3 Centro de Investigación Biomédica en Red Fisiopatología de la Obesidad y Nutrición (CIBERobn), Instituto de Salud Carlos III, 28029 Madrid, Spain; 4 Navarra Institute for Health Research (IdiSNa), 31008 Pamplona, Spain; 5 Department of Bioinformatics, Making Genetics S.L., 31002 Pamplona, Spain; mlmansego@making-genetics.eu; 6 Precision Nutrition and Cardiometabolic Health Program, Madrid Institute for Advanced Studies (IMDEA), IMDEA Food, 28049 Madrid, Spain

**Keywords:** waist circumference, epigenetics, DNA methylation

## Abstract

Epigenetic signatures such as DNA methylation may be associated with specific obesity traits in different tissues. The onset and development of some obesity-related complications are often linked to visceral fat accumulation. The aim of this study was to explore DNA methylation levels in peripheral white blood cells to identify epigenetic methylation marks associated with waist circumference (WC). DNA methylation levels were assessed using Infinium Human Methylation 450K and MethylationEPIC beadchip (Illumina) to search for putative associations with WC values of 473 participants from the Methyl Epigenome Network Association (MENA) project. Statistical analysis and Ingenuity Pathway Analysis (IPA) were employed for assessing the relationship between methylation and WC. A total of 669 CpGs were statistically associated with WC (FDR < 0.05, slope ≥ |0.1|). From these CpGs, 375 CpGs evidenced a differential methylation pattern between females with WC ≤ 88 and > 88 cm, and 95 CpGs between males with WC ≤ 102 and > 102 cm. These differentially methylated CpGs are located in genes related to inflammation and obesity according to IPA. Receiver operating characteristic (ROC) curves of the top four significant differentially methylated CpGs separated by sex discriminated individuals with presence or absence of abdominal fat. ROC curves of all the CpGs from females and one CpG from males were validated in an independent sample (*n* = 161). These methylation results add further insights about the relationships between obesity, adiposity-associated comorbidities, and DNA methylation where inflammation processes may be involved.

## 1. Introduction

Obesity has increased worldwide to reach epidemic proportions [1]. According to the World Health Organization, the prevalence of obesity nearly doubled between 1980 and 2014, reaching an alarming percentage of 11% in men and 15% in women worldwide [2]. This rising burden is accompanied by the increased prevalence of obesity-linked comorbidities, such as cardiovascular diseases, diabetes, cancer, and musculoskeletal disorders, among others [2]. The development of these obesity-related complications are more often associated with visceral fat instead of total body fat [3]. In this context, waist circumference (WC) measurements can be a useful tool to assess abdominal obesity, anatomically discriminating between visceral and subcutaneous fat depositions in individuals with obesity [4].

Obesity is a multifactorial disease resulting from the interaction of environmental and lifestyle factors with the genetic make-up [5]. Genetic variations contribute to the individual susceptibility to developing diseases, but accumulating evidences suggest that epigenetic phenomena are also involved [6]. Epigenetic marks and modifications may play a causative role in the development of obesity by altering the expression of obesity-related genes, or alternatively, be a consequence of obesity, predisposing some subjects to associated comorbidities [7]. Indeed, several epigenome-wide association studies (EWASs) have revealed that obesity traits are associated with DNA methylation shifts in different tissues [6,8,9]. However, most of these studies use body mass index (BMI) instead of WC, but WC appears to be a more accurate and convenient surrogate for central obesity assessment [4], being therefore more associated with obesity-related diseases [3]. Indeed, there are only few EWAS analyzing the association of methylation status and WC [10,11,12,13,14,15,16]. 

The aim of the current research was to explore DNA methylation levels in peripheral white blood cells to identify epigenetic methylation signatures associated with WC. 

## 2. Subjects and Methods

### 2.1. Participants

This study was performed in 523 adult participants from cohorts composing the Methyl Epigenome Network Association (MENA) project such as DiOGenes-UNAV with *n* = 58 [17], OBEPALIP with *n* = 29 [18], Food4Me-UNAV with *n* = 42 [19], GEDYMET with *n* = 57 [20], ICTUS with *n* = 7 [21], NUGENOB-UNAV with *n* = 42 [22], PREDIMED-UNAV with *n* = 129 [23], RESMENA with *n* = 47 [24], OBEKIT with *n* = 100 [25], and NormoP with *n* = 12 [26], whose providers are gratefully acknowledged. Study design, characteristics, and exclusion and inclusion criteria of each of these cohorts have been previously described. All of these studies were approved by the research ethics committees at all recruiting centers in compliance with the Helsinki Declaration of ethical principles for medical research involving human subjects (DiOGenes KF01-267787 IHE 4-1-2.0091 dd. 23-03-2006, OBEPALIP 007/2009, Food4Me-UNAV 041/2012, GEDYMET 14-281, ICTUS 2/10, NUGENOB-UNAV 5/04/2001, PREDIMED-UNAV 50/2005, RESMENA 065/2009, OBEKIT and NormoP 132/2005). All participants provided written informed consent. 

A validation was performed in an independent sample of individuals (*n* = 161) from the OBEKIT and NormoP studies (not included in the MENA study) (Supplementary Table S1).

### 2.2. Study Variables

Anthropometric measurements at baseline were obtained from databases of the aforementioned cohorts following standardized validated protocols [27]. BMI was calculated dividing weight in kg by height in meters squared (kg/m^2^). WC was measured at the mid-point between the lower rib and the iliac crest using a tape measure. Blood samples for methylation analysis were obtained at the same visit. A summary of the studies design and collected samples are reported (Supplementary Table S2).

### 2.3. DNA Extraction, DNA Methylation Analysis, and Treatment of Methylation Raw Data

Procedures explaining DNA extraction and DNA methylation analysis have been detailed elsewhere [26]. Briefly, Infinium Human Methylation 450K bead chip technology (Illumina, San Diego, CA, USA) was employed to measure DNA methylation levels in all the cohorts, except OBEKIT, which was performed with Infinium MethylationEPIC beadchip (Illumina). 

### 2.4. Treatment of Methylation Raw Data

Beta-values were employed to assess methylation levels in order to estimate the methylation degree using the ratio of the methylation probe intensity and the overall intensity, corresponding to the percentage of methylation on a specific site [28]. Intensity data were obtained using the ChAMP package for R v.1.11.0 [29] as described elsewhere [30]. Then, the filtering process was performed in probes with a detection *p*-value > 0.01 in one or more samples, probes with a beadcount < 3 in at least 5% of samples, non-CG site (CpG) probes, probes with single nucleotide polymorphisms [31], probes that align to multiple locations [31], and probes located on the X or Y chromosomes. 

Out of the 523 initial participants, 20 samples with a failed CpG fraction above 0.01 were eliminated, leaving 503 individuals. Afterwards, intra-cell type normalization using the subset-quantile within array normalization method was performed to avoid any bias introduced by the Infinium type 2 probe design [32]. Then, multidimensional scaling plots based on top 1000 most variable probes were carried out to evaluate the similarity of normalized methylation samples in both batches and the pooled data. A total of 29 samples failed to accomplish this requirement, leaving 474 participants for the subsequent analyses.

Since the methylation data was obtained at different times in the lab, following subset-quantile within array normalization, the ComBat normalization method was used to assess and correct the magnitude of batch effects [33,34]. Furthermore, the Houseman procedure [35] was employed to correct differences in methylation resulting from cellular heterogeneity.

The data have been deposited in NCBI’s Gene Expression Omnibus [36] and are accessible through GEO Series accession number GSE115278 [37].

### 2.5. Statistical Analysis

After pre-processing, the linear models for microarray data (LIMMA) package for the R statistical software [29] was used to compute a linear regression between DNA methylation and WC (*n* = 473, since data of WC was not available for one participant) adjusted by the effect of confounding factors, such as sex, age, study, and batch effect. Raw *p*-values were corrected employing the Benjamini–Hochberg procedure as a correction for multiple comparisons. A quantile–quantile plot showed that there was a small inflation of the adjusted *p*-values, which were corrected [38] (Supplementary Figure S1). The statistically significant threshold for CpG selection was set by a false discovery rate cut-off of 0.05 and a slope ≥ |0.1|.

In order to ascertain if there were methylation differences between subjects with high and low WC, and due to gender differences in the established cut points for WC, the selected CpGs were categorized by sex. WC was used as an anthropometric marker of abdominal obesity and was dichotomized following the National Cholesterol Education Program Adult Treatment Panel III. The cut-off for WC in females was 88 cm, whereas, a WC of 102 cm was considered for males [39]. Differentially methylated CpGs were explored using Student’s *t*-test with the Bonferroni correction (in order to avoid type I errors) between females with WC ≤ 88 and > 88 cm, and between males with WC ≤ 102 and > 102 cm. A *p*-value < 7.47·10^–5^ was considered statistically significant. Receiver operating characteristic (ROC) curves adjusted by age were calculated for the top four significant differentially methylated CpGs for each sex. ROC curves were validated in an independent sample of individuals (*n* = 161).

Statistical calculations were performed with Stata version 12.1 (StataCorp 2011, College Station, TX, USA), unless otherwise indicated. Correlation graphs and box plots were generated using GraphPad Prism 6 (Graph-Pad Software, San Diego, CA, USA). The volcano plot, quantile–quantile plot, and heat map (using library gplots and the heatmap.2 function) were created with the R software [29].

### 2.6. Ingenuity Pathway Analysis

The genes corresponding to the differentially methylated CpGs between females with WC ≤ 88 and > 88 cm, or males with WC ≤ 102 and > 102 cm, were analyzed by Ingenuity Pathway Analysis (IPA) software (Qiagen Redwood City, CA, USA, www.ingenuity.com). Associated pathways and gene regulatory networks were identified by predefined pathways and functional categories of the Ingenuity Knowledge Base [40]. Canonical pathway analyses were performed with IPA’s core analysis module and selected if *p* < 0.05 after Fisher’s test as statistically significant.

## 3. Results

Anthropometric characteristics including weight, BMI, and WC of the participants are reported (Table 1). The proportion of females is higher than men and the average age is 47 years, although there are studies with a mean of 27 years, such as GEDYMET or 65 years, such as PREDIMED-UNAV. Furthermore, mean BMI indicates that the average population is obese, but the range of means of the studies goes from 22.8 to 44.3 kg/m^2^. WC values showed a higher number of individuals from both sexes with central adiposity, although it also depends on the study. 

**Figure 1:**
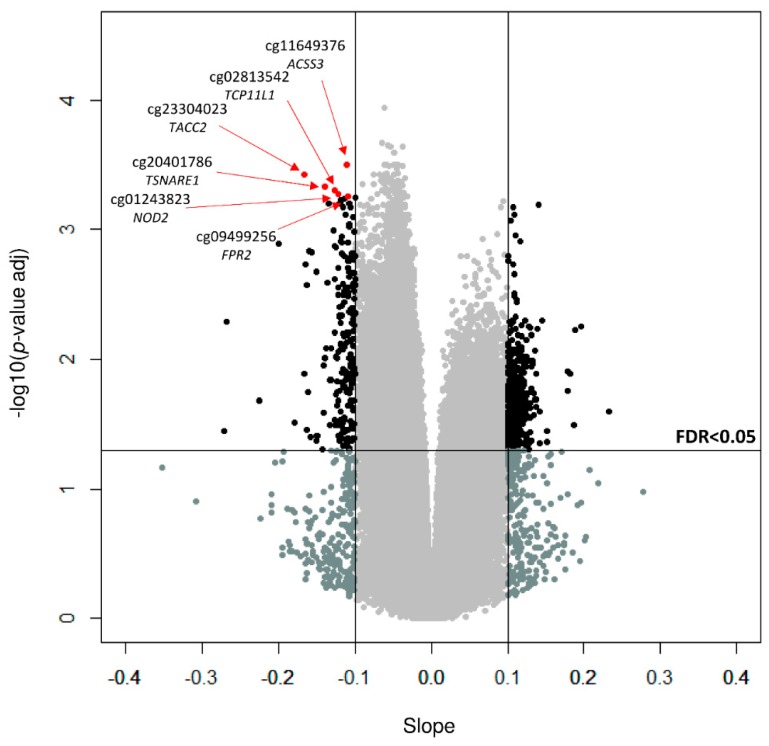
Volcano plot of waist circumference-associated CpGs (corresponding gene according to Illumina). Points above the horizontal line showed a false discovery rate (FDR) < 0.05 and outside the vertical lines represented a slope ≥ |0.1|.

**Figure 2:**
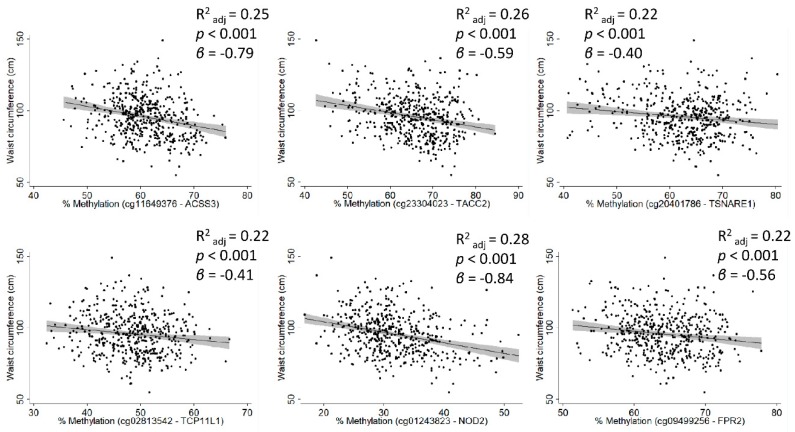
Linear regression graphs adjusted by sex and age representing the association between waist circumference and methylation β values of top six CpGs (corresponding gene according to Illumina) selected by a slope ≥ |0.1| and false discovery rate < 0.05. The grey stripe represent a 95% confidence band.

Linear regressions (LIMMA) between methylation values and WC were performed and 669 significant CpGs were selected after applying false discovery rate cut-off of 0.05 and a slope ≥ |0.1|, and correcting for inflation (Supplementary Table S3). The six top CpGs were cg11649376 (corresponding gene according to Illumina CG database *ACSS3*), cg23304023 (*TACC2*), cg20401786 (*TSNARE1*), cg02813542 (*TCP11L1*), cg01243823 (*NOD2*), and cg09499256 (*FPR2*), which are plotted in Figure 1. Linear regression graphs adjusted by sex and age between methylation values and WC for these six CpGs are depicted in Figure 2. 

Participants were classified according to sex and WC, separating females with WC ≤ 88 and > 88 cm, and males with WC ≤ 102 and > 102 cm, in order to analyze whether methylation was differential between both groups for each sex. There were 121 females with WC ≤ 88 cm and 182 with WC > 88 cm, and 82 males with WC ≤ 102 cm and 88 with WC > 102 cm (Table 1). Methylation values of the 669 CpGs were compared between both WC groups for each sex. For females, 375 CpGs showed statistically significant differences and 95 CpGs for males (*p* < 7.47×10^−5^) (Supplementary Table S4A and S4B, respectively).

**Figure 3:**
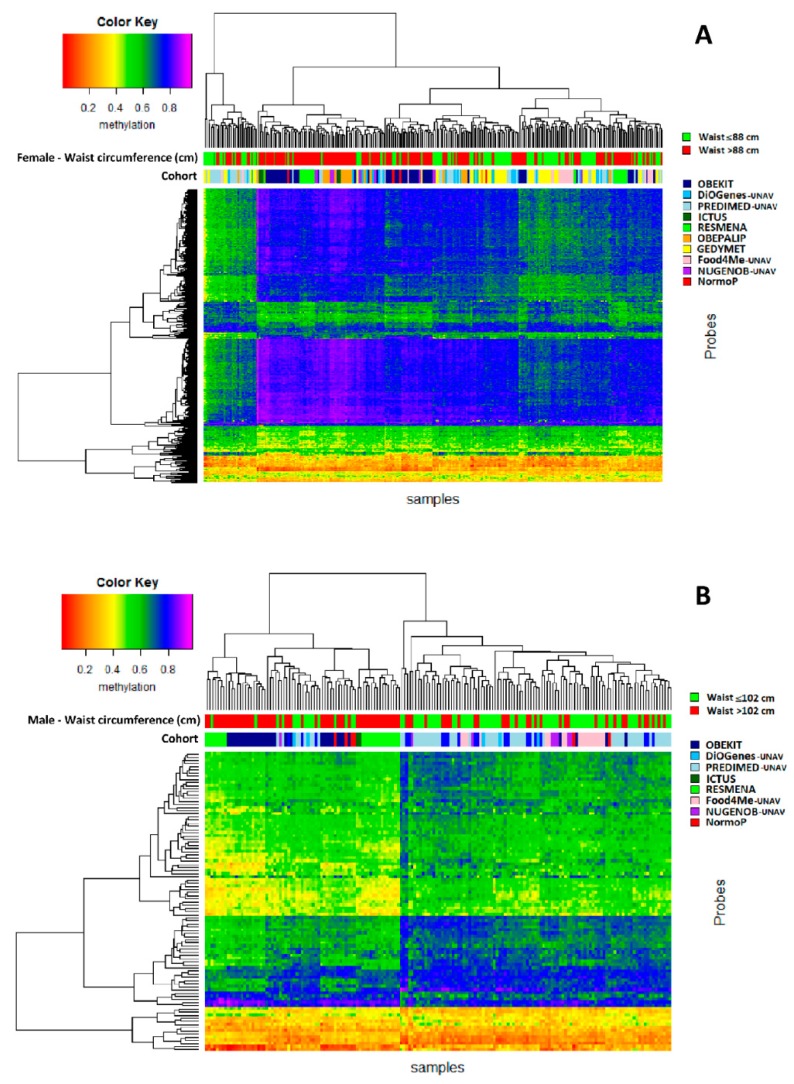
Heat maps representing the clusters between methylation levels (rows) and subjects from the different cohorts (columns). (**A**) Heat map of 375 CpGs selected by Student’s *t*-test between females with waist circumference ≤ 88 and > 88 cm. (**B**) Heat map of 95 CpGs selected by Student’s *t*-test between males with waist circumference ≤ 102 and > 102 cm. Significance: *p* < 7.47 × 10^–5^ after Bonferroni correction.

The respective 375 and 95 CpGs were clustered in a heat map according to methylation patterns (Figure 3). For females (Figure 3A), three main clusters of 35, 84, and 184 individuals were generated. The first and third clusters contained 51.1% of individuals with WC > 88 cm. However, the second cluster included 83.3% of WC > 88 cm. On the other hand, males (Figure 3B) were clustered in two groups of 71 and 99 individuals. The first cluster group showed a percentage of 77.5% of males with WC > 102 cm, while in the second group, the percentage was 33.3%. The difference in WC proportions of the clusters was statistically significant (*p* < 0.001) for both sexes. 

Canonical pathways were obtained from IPA for the significant 375 and 95 CpGs for females and males, respectively (Supplementary Figure S2). Some of the statistically significant pathways were related to inflammation and obesity in both sexes. In females, some of these pathways were lipoate biosynthesis and incorporation II, role of *JAK2* in hormone-like cytokine signaling, inflammasome pathway, growth hormone signaling, and acetate conversion to acetyl-CoA. In the case of males, some of the pathways were inflammasome pathway, *TREM1* signaling, intrinsic prothrombin activation pathway, pentose phosphate pathway, Gai signaling, *STAT3* pathway, and *GP6* signaling pathway. 

The top four CpGs showing differences between WC ≤ 88 or > 88 cm in females were cg09907509 (*c13orf36*), cg17478979 (*ZC3H12D*), cg24679890 (*MYO9B*), and cg06638795 (*KCNG3*) (Supplementary Table S4A). In males, the top four CpGs with differences between WC ≤ 102 or > 102 cm were cg01807303 (NA), cg03325085 (*JPH3*), cg02813542 (*TCP11L1*), and cg16379885 (*GRIK3*) (Supplementary Table S4B). 

**Figure 4:**
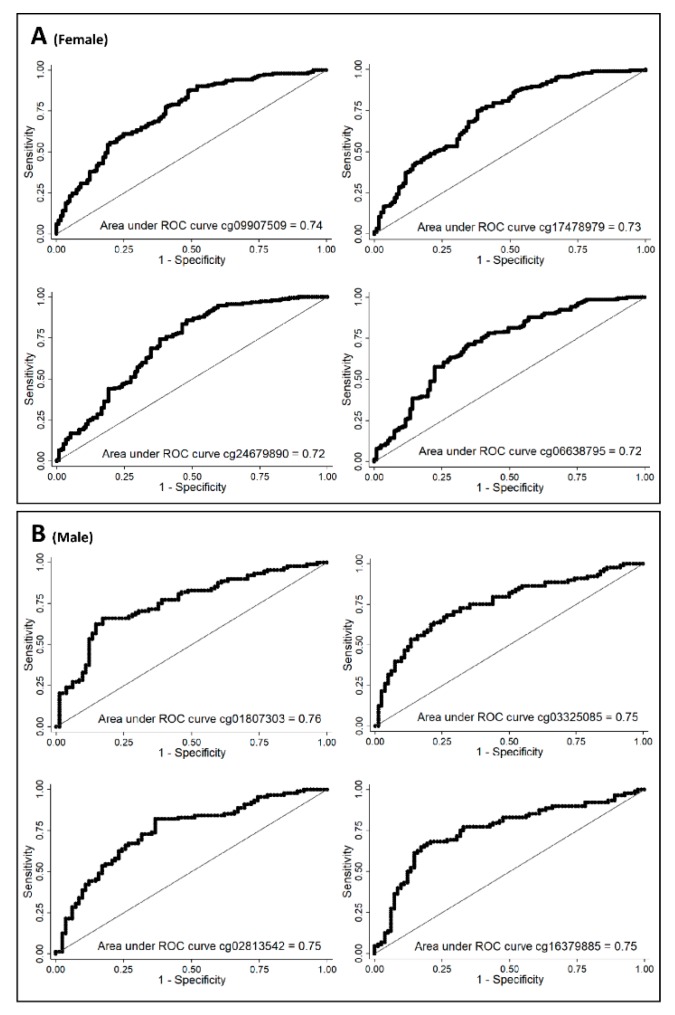
Receiver operating characteristic (ROC) curves. (**A**) Top four significant differentially methylated CpGs for females adjusted by age; (**B**) Top four significant differentially methylated CpGs for males adjusted by age.

In order to analyze whether the four CpGs were valuable for assessing WC, areas under the curve (AUC) of the ROC curve were calculated for each CpG. For females, the AUCs were cg09907509 = 0.74, cg17478979 = 0.73, cg24679890 = 0.72, and cg06638795 = 0.72 (Figure 4A). For males, the AUCs were cg01807303 = 0.76, cg03325085 = 0.75, cg02813542 = 0.75, and cg16379885 = 0.75 (Figure 4B). Validation of these results was performed in an independent sample (*n* = 161). Results showed valuable AUCs (AUC > 0.70) for the four CpGs in females, confirming the results of the current investigation, whereas only cg02813542 was validated in males (Table 2).

## 4. Discussion

This study involving the MENA project reports the association between DNA methylation and WC in specific CpGs of several genes, mainly related to inflammation, obesity, and related comorbidities. Furthermore, current analyses showed the differential pattern in methylation depending on the degree of abdominal fat in both sexes, which can be predicted by several CpGs with AUCs > 0.70. This assay adds further insights into the relationship between obesity, associated comorbidities, and epigenetic DNA methylation. 

Obesity is usually classified according to BMI, which is an index widely employed for measuring the overall body size or generalized adiposity [12]. However, WC has been suggested as a better estimator of metabolic complications accompanying excessive adiposity [1], being associated with metabolic diseases, such as type 2 diabetes (T2D) and cardiovascular events [1]. Indeed, few EWAS have studied the putative association between DNA methylation and WC values [10,11,12,13,14,15,16], in contrast to the number of EWAS correlating methylation with BMI. In this sense, we found a relationship between DNA methylation levels of 669 CpGs and WC (slope ≥ |0.1| and false discovery rate < 0.05). The top six CpGs corresponded to the genes *ACSS3, TACC2, TSNARE1, TCP11L1, NOD2*, and *FPR2* (according to the Illumina CG database). Interestingly, methylation of all these genes was also associated with BMI (data not shown), indicating that both general and central adiposity might be related to epigenetic modifications in these genes [41]. Moreover, in a subanalysis (*n* = 108) concerning the current study, WC was associated with circulating levels of TNF-α and C-reactive protein (CRP), and some of the selected CpGs were also related to these inflammatory molecules (data not shown). These associations confirm that inflammatory processes are involved in the obesity status as previously described [41], which may be partly mediated by epigenetic mechanisms. 

Remarkably, five of the six selected genes have been found related to different traits in previous EWAS. For instance, BMI has been associated with methylation in CpGs of the genes *FPR2*, *TACC2*, *TSNARE1*, *TCP11L1,* and *NOD2* [42]. Interestingly, the selected CpG cg01243823 (*NOD2*) from this study was related to BMI in different investigations [9,42,43,44]. Moreover, methylation in some CpGs located in the genes *TACC2, TSNARE1, TCP11L1*, and *NOD2,* including the CpGs cg02813542 (*TCP11L1*) and cg01243823 (*NOD2*) selected in this study, was statistically different when comparing diabetic and non-diabetic subjects [45]. Furthermore, the CpG cg02813542 was associated with BMI in adipose tissue from non-diabetic subjects, and the CpG cg01243823 was related to fasting glucose in the same samples [45]. Other CpGs in the genes *NOD2* [46] and *TCP11L1* [47] have been linked to atherosclerosis, and in the *TSNARE1* gene, with CRP [48]. The inflammation-related molecule CRP was also connected to the CpG cg01243823 selected in this research [48]. 

Although DNA methylation in the selected CpGs might not be exerting changes in the expression of the genes where they are located, their involvement in metabolic and inflammatory processes might indicate a putative connection. Specifically, the gene *ACSS3* encodes a protein that converts propionate to propionyl-CoA allowing it to enter mitochondrial respiration and the Krebs cycle [49]. Proteins of the same family (acyl-CoA synthases) have been related to inflammatory processes [50,51]. The gene *TACC2* has been found hypomethylated before weight loss in the top 20 differentially methylated CpGs in a study comparing before and after weight loss [52]. In the case of *TSNARE1*, differential methylation levels between basal and insulin-stimulated muscle has been described [53], as well as the association between methylation degree and C-reactive protein, which is a sensitive marker for low-grade inflammation [48]. The *NOD2* gene has been related to inflammation, since a higher expression of *NOD2* may contribute to an increased inflammatory response of immune cells in diet-induced obesity [54]. Genetic variants of *NOD2* may also influence the risk of chronic inflammation, insulin resistance, and T2D [55]. The gene *FPR2* binds to lipid mediators such as resolvin D1 for promoting resolution of inflammation [56], including the resolution of obesity-induced chronic low-grade inflammation [57]. These lipid mediators have been found decreased in obese mice [58]. In the case of the *TCP11L1* gene, two SNPs (rs3168277 and rs2273553) showed significant association with fasting blood glucose and HDL-C, respectively [59]. 

Abdominal obesity is a major risk factor concerning systemic inflammation, hyperlipidemia, insulin resistance, and cardiovascular disease [60]. Our study revealed that individuals with abdominal obesity traits (WC females > 88 cm, males > 102 cm) exhibited a differential methylation pattern for at least 375 and 95 CpGs, respectively. Remarkably, more than 75% of the males and more than 80% of the females that were in the same cluster with similar methylation patterns presented abdominal obesity. Therefore, specific epigenetic modifications may be representative for people with central obesity and might potentially influence the onset and development of other adiposity-associated complications, such as T2D and cardiovascular events. Indeed, these differentially methylated CpGs were related to pathways of inflammation, obesity, and related comorbidities for both males and females, such as the pathway lipoate biosynthesis and incorporation II, since lipoic acid has anti-inflammatory and anti-oxidant actions, and it has been reported to be helpful against insulin resistance and hypertriglyceridemia [61]. Additionally, the role of *JAK2* in the hormone-like cytokine signaling pathway, where *JAK2* is involved in binding to receptors such as *GHR*, activating the signal transduction cascade, and growth hormone signaling. The expression of *GHR* has been found to be increased in adipose tissue abdominal depots, compared to gluteal depots, suggesting an effect of growth hormone to specifically reduce abdominal adipose tissue mass [62]. Moreover, growth hormone secretion is usually impaired in obesity [63]. Furthermore, in the inflammasome pathway, inflammasomes play the role of central regulators connecting the induction and the progression of autoinflammatory disease with cellular stress from obesity-induced inflammation, metabolic distress, and other stress signals [64]. The *TREM-1* gene in TREM1 signaling acts as an amplifier of inflammation expressed in macrophages. *TREM-1* has been found to be overexpressed in patients with obesity, predisposing pre-diabetics to obesity-induced insulin resistance [65]. The rate-limiting enzyme in the pentose phosphate pathway, glucose-6-phosphate dehydrogenase (G6PD), is an important mediator of adipose tissue inflammation and insulin resistance in subjects with obesity [66]. In the case of the intrinsic prothrombin activation pathway, prothrombin activity is higher in individuals with central obesity [67], and weight loss interventions reduce the circulating levels of prothrombic markers [68].

The top four CpGs that were found to be differentially methylated between individuals with and without abdominal obesity were located at the genes *c13orf36*, *ZC3H12D*, *MYO9B*, and *KCNG3* in females; and *JPH3*, *TCP11L1*, and *GRIK3* in males (one CpG had no annotated gene). As aforementioned, the observed DNA methylation differences might not be indicative of changes in the corresponding gene expression, but their relationship with obesity and other metabolic processes might suggest a possible link. For instance, the gene *ZC3H12D* is involved in inflammatory diseases and in immune response [69]. CpG cg24679890 (*MYO9B*) has been previously related to BMI in various EWAS [16]. In the case of *JPH3*, it is necessary for glucose-stimulated insulin secretion [70]. Additionally, the gene *GRIK3*, involved in glutamate receptor signaling, was one of the genes differentially methylated related to inflammation and T2D in siblings born before and after maternal bariatric surgery [71]. Interestingly, cg02813542 (*TCP11L1*) also appeared in the top six CpGs after the association between DNA methylation and WC, as previously mentioned. Nevertheless, the *c13orf36* and *KCNG3* genes have not already been described in relation to obesity.

Since the methylation patterns of some CpGs were able to characterize individuals according to whether they do or do not have abdominal obesity, the ability of the four CpGs with higher AUC to differentiate between both WC groups for each sex was analyzed. All the CpGs distinguished WC groups with AUCs above 0.70. However, validation in an independent sample showed that cg09907509-*c13orf36*, cg17478979-*ZC3H12D,* cg24679890-*MYO9B*, and cg06638795-*KCNG3* for females, and cg02813542-*TCP11L1* for males were capable of discriminating individuals regarding their central adiposity. Although these CpGs cannot be confirmed as a cause or consequence of central adiposity, they might be considered for futures studies as potential biomarkers for early prediction or putative targets for the development of therapeutic approaches in the prevention and treatment of diseases, such as obesity or associated comorbidities.

The present study presents some limitations. The most important is the cross-sectional nature of the study, which does not allow the establishment of causality. Longitudinal studies are necessary to evaluate the direction of the association between DNA methylation and WC, although a validation in an independent population (*n* = 161) confirmed the general trends. In addition, RNA assays would have been interesting to relate methylation to gene expression. Another limitation is that methylation is tissue-specific [72]. The ideal tissue for this study would have been visceral adipose tissue, but peripheral blood is the best non-invasive alternative tissue that reflects multiple metabolic and inflammatory pathways. Indeed, multiple studies have analyzed DNA methylation in blood in relation to obesity [9,10,73] and inflammation [74,75]. Furthermore, some studies have demonstrated that blood can reflect epigenetic changes in other tissues, such as adipose tissue [73,76]. Finally, though multiple comparison tests and statistical adjustments for potential confounding factors such as sex, age, cohorts, bead chips, and cell composition heterogeneity were performed, type I and type II errors could not be discarded. However, correction of inflation was performed to avoid type I errors. Despite these limitations, we understand that our study contributes to the growing body of evidence in support of epigenetic methylation mechanisms correlating with obesity features in a relative wide sample (*n* = 473), followed by a validation (*n* = 161).

In conclusion, this study found associations between DNA methylation in several CpGs and an excessive central adiposity in peripheral white blood cells. In addition, novel loci with differential DNA methylation variations in individuals with abdominal adiposity were identified. Further studies, especially longitudinal studies, are needed to assess causality and identify additional putative methylation biomarkers and early predictors of obese phenotypes.

## Figures and Tables

**Table 1:** Anthropometric, clinical, and biochemical characteristics of the study population categorized by project/consortium.

	TOTAL		ADULTS (*n* = 474)																			
			DiOGenes-UNAV		OBEPALIP		Food4Me-UNAV		GEDYMET		ICTUS		NUGENOB-UNAV		PREDIMED-UNAV		RESMENA		NormoP		OBEKIT	
Variables	*n*	Values	*n*	Values	*n*	Values	*n*	Values	*n*	Values	*n*	Values	*n*	Values	*n*	Values	*n*	Values	*n*	Values	*n*	Values
Sex (females)	474	303 (64)	52	27 (52)	29	29 (100)	39	21 (54)	57	57 (100)	7	5 (71)	22	14 (64)	116	59 (51)	44	22 (50)	12	6 (50)	96	63 (66)
Age (years)	474	47.0 (14.3)	52	42.7 (5.8)	29	37.4 (7.3)	39	41.7 (10.0)	57	27.0 (6.2)	7	57.1 (7.4)	22	34.7 (9.7)	116	65.0 (3.7)	44	48.6 (10.1)	12	39.4 (5.6)	96	46.8 (9.6)
Weight (kg)	474	81.7 (19.1)	52	95.3 (17.7)	29	83.1 (9.5)	39	74.4 (14.6)	57	60.7 (8.8)	7	121.9 (15.2)	22	87.3 (20.8)	116	71.7 (9.2)	44	103.0 (18.1)	12	65.8 (9.3)	96	89.2 (13.6)
BMI (kg/m^2^)	474	30.0 (5.7)	52	33.9 (3.8)	29	31.6 (3.1)	39	26.0 (5.3)	57	24.1 (3.5)	7	44.3 (4.0)	22	31.1 (8.2)	116	27.7 (2.3)	44	36.5 (3.7)	12	22.8 (1.5)	96	31.9 (3.7)
Waist circumference (cm)	473	95.8 (16.1)	52	107.5 (11.5)	29	95.4 (6.8)	39	87.9 (12.4)	57	72.7 (7.9)	7	125.3 (11.1)	22	93.7 (19.4)	115	91.8 (8.2)	44	112.5 (12.4)	12	78.2 (7.5)	96	104.1 (10.5)
Female ≤ 88 (cm)	121	76.3 (7.8)	0	NA	2	81.6 (3.2)	14	77.5 (7.4)	55	71.9 (6.7)	0	NA	5	72.0 (4.6)	35	82.3 (4.9)	0	NA	6	74.4 (8.0)	4	85.1 (3.0)
Female > 88 (cm)	182	100.9 (10.0)	27	102.9 (9.1)	27	96.4 (5.8)	7	97.9 (10.0)	2	95.0 (1.4)	5	120.6 (8.9)	9	102.9 (10.7)	24	92.8 (3.5)	22	105.7 (10.7)	0	NA	59	102.0 (9.7)
Male ≤ 102 (cm)	82	92.8 (7.4)	5	97.6 (3.0)	0	NA	16	90.1 (9.2)	0	NA	0	NA	5	81.0 (4.4)	47	95.6 (4.5)	1	94.0 (NA)	6	82.0 (4.8)	2	95.4 (4.0)
Male > 102 (cm)	88	114.8 (10.0)	20	116.0 (10.5)	0	NA	2	109.0 (8.5)	0	NA	2	137.2 (5.4)	3	123.2 (13.0)	9	105.6 (2.6)	21	120.5 (8.5)	0	NA	31	111.0 (7.2)

Values are mean (SD), except for sex, which is represented as number of cases (%). BMI: body mass index; NA: not applicable.

**Table 2:** Validation of ROC curves in an independent sample (*n* = 161) for the top four significant differentially methylated CpGs for each sex adjusted by age.

CpG	AUC
**Women**	
cg09907509	0.73
cg17478979	0.77
cg24679890	0.72
cg06638795	0.72
**Men**	
cg01807303	0.63
cg03325085	0.60
cg02813542	0.71
cg16379885	0.62

## Supplementary Materials

The following are available online at https://www.mdpi.com/2073-4425/10/6/444/s1: Figure S1: Q–Q plot of corrected adjusted *p*-values, Figure S2: Canonical pathways from Ingenuity Pathway Analysis. A) Canonical pathways from 375 CpGs selected by Student’s *t*-test between females with waist circumference ≤ 88 and > 88 cm. B) Canonical pathways from 95 CpGs selected by Student’s *t*-test between males with waist circumference ≤ 102 and > 102 cm. Significance: *p* < 7.47·10^–5^ after Bonferroni correction, Table S1: Anthropometric, clinical and biochemical characteristics of the validation population, Table S2: Summary of the studies and the collected anthropometric and DNA methylation measurements, Table S3: Selection of 669 CpGs applying false discovery rate cut-off of 0.05 and a slope ≥ |0.1|, Table S4: Selection of CpGs separating by waist circumference cut-points. (A) Selection of 375 CpGs for females after comparing waist circumference ≤ 88 and > 88 cm. (B) Selection of 95 CpGs for males after comparing waist circumference ≤ 102 and > 102 cm.
